# Total Phenolic Content and Antimicrobial Activity of Different Lithuanian Propolis Solutions

**DOI:** 10.1155/2013/842985

**Published:** 2013-03-20

**Authors:** Kristina Ramanauskienė, Asta Marija Inkėnienė, Vilma Petrikaitė, Vitalis Briedis

**Affiliations:** ^1^Department of Clinical Pharmacy, Lithuanian University of Health Sciences, A. Mickevičiaus 9, 44307 Kaunas, Lithuania; ^2^Department of Pharmaceutical Chemistry, Lithuanian University of Health Sciences, A. Mickevičiaus 9, 44307 Kaunas, Lithuania

## Abstract

The manufacture of ethanol-free propolis solutions offers a broader application. A few trials with Lithuanian propolis have been conducted. The aims of the study are to manufacture propolis water and water-free solutions and evaluate the quality and antimicrobial activity of these solutions. The studied solutions containing 2.5%, 5%, and 10% propolis are prepared. As solvents, purified water, 70% v/v ethanol, 96.3% v/v ethanol, propylene glycol, and their systems were used. Determination of total levels of phenolic compounds (FAE mg/g) is based on colour oxidation-reduction reaction using Folin-Ciocalteu reagent under alkaline conditions and performed at 765 nm wavelength using UV spectrophotometer. The highest content of phenolic compounds was determined in solutions containing 10% propolis extracts, and the lowest amounts in 2.5% propolis extracts. The water extracted the lowest amount of phenolic compounds from crude propolis, ethanol extracted the highest amount, and propylene glycol ranked the middle position. It is determined that technological parameters (stirring, temperature) contribute to content of phenolic compounds. During microbiological study, MICs were determined. The studies showed that water extracted propolis solutions and solvents mixture did not inhibit the growth of the studied microorganisms, and propolis solutions in propylene glycol were found to have antimicrobial activity.

## 1. Introduction

Propolis as an active substance is attractive due to its antimicrobial and antimycotic properties and as a natural substance whose effect was proven by biological experiments [1–3]. Raw propolis is composed of 50% resin, containing flavonoids (flavones: chrysin, apigenin, and luteolin; flavanols: rutin, morin, quercetin, myricetin, kaempferol, quercitrin, and galangin; flavanones: naringin, (±)-naringenin, and hesperitin; isoflavones: daidzein and genistein), phenolic acids (caffeic, cinnamic, p-coumaric, ferulic, p-hydroxybenzoic, gallic, etc.) and their esters, 30% wax, 10% essential oils, 5% pollen and 5% terpenoids, steroids, and amino acids and other organic compounds [4]. The composition of propolis depends on the vegetation at the site of collection. Propolis has been used extensively in folk medicine for many years, and there is substantial evidence indicating that propolis has antiseptic, antifungal, antibacterial, antiviral, anti-inflammatory, antioxidant, immunomodulatory, and antitumor properties [5–7]. The antibacterial, antiviral, and antifungal activities are the most popular among the most extensively investigated biological actions of propolis [8–10]. Propolis is one of the most potent natural antibiotics characterized by a very wide spectrum of effects. Its therapeutic application does not induce germ resistance and does not destroy useful microflora [7]. Propolis has a fungicidal effect on a number of species of fungi, including *Candida albicans, Aspergillus niger, Botrytis cinerea, Ascosphaera apis, *and* Plasmopara viticola* [11].

Current applications of propolis include over-the-counter preparations for cold syndrome (upper respiratory tract infections, common cold, and flu-like infection) as well as dermatological preparations useful in wound healing, treatment of burns, acne, herpes simplex and genitalis, and neurodermatitis [12]. Propolis can also be used for dental disease prevention and treatment. It is found that propolis has a strong antimicrobial activity against *Streptococcus mutans*,* Streptococcus sobrinus*,* Streptococcus sanguinis*, and* Candida albicans*, which are important for oral pathogens. Most preparations are based on ethanolic extracts of propolis.

The manufacture of ethanol-free propolis solutions offers a broader application in medicine and everyday use. There are many data on chemical composition of propolis solutions in ethanol and antimicrobial activity. However, a few trials with Lithuanian propylene glycol extracted propolis, its water solutions, and appropriate solvent mixtures have been conducted.

The aims of the study are to manufacture propolis water and water-free solutions and evaluate quality as well as antimicrobial activity of these solutions.

## 2. Material and Methods 

### 2.1. Manufacture of Propolis Solutions

The studied solutions containing 2.5%, 5%, and 10% propolis 200 mL are prepared. As solvents, purified water, 70% v/v ethanol, 96.3% v/v ethanol, propylene glycol (1,2-propanediol), and their systems composing of 6.25 mL 96.3% w/w ethanol, 2.5 g propylene glycol, and purified water up to 25 mL of total volume are used. Crushed crude propolis is soaked in an appropriate amount of solvent and left for maceration for seven days [13, 14]. Manufactured extraction is filtered using paper filter.

### 2.2. Determination of Total Phenolic Compounds Content

Determination of total levels of phenolic compounds is based on colour-oxidation-reduction reaction using Folin-Ciocalteu reagent under alkaline conditions and performed at 765 nm wavelength using Unicam Helios *α* UV spectrophotometer (Unicam, Cambridge, UK). Total amounts of phenolic compounds are expressed as ferulic acid equivalent (FAE) mg/g.

Colour reaction: into 100 mL measurement flask 15 mL of purified water, 4 mL of Folin-Ciocalteu reagent depending on concentration and studied solution, and then 6 mL of 20% sodium bicarbonate are added. Diluted with purified water up to 100 mL measurement. The manufactured solution is stored for 2 h at room temperature for reaction to take place. Absorbtion is measured by reference solution using purified water [15, 16].

### 2.3. Determination of Microbiological Activity

The study is performed following the Ph. Eur. 01/2002, 2.6.12. Microbiological study is conducted under aseptic conditions. During microbiological study, MIC (minimum inhibitory concentration)—the highest dilution of preparation (the lowest concentration of preparation), which inhibits a certain growth of standard microorganism culture was determined. When the main solutions were prepared, dilutions were performed with 10 mL of Mueller-Hinton agar (Mueller-Hinton Agar, Becton, Dickinson and Company) to obtain working solutions in Mueller-Hinton agar, in which MIC effect of the studied preparations on the growth of standard microorganisms was determined. Then, every Petri dish containing dilutions and covered with Mueller-Hinton agar was inoculated with standard bacteria: *Staphylococcus aureus, Klebsiella pneumoniae, Escherichia coli, Pseudomonas aeruginosa, Enterococcus faecalis, Proteus mirabilis, Bacillus cereus, Bacillus subtilis, *and* Candida albicans*. Cultures were incubated for 24 h in thermostat at 37°C temperature, and then the growth of microorganisms in the zone of inoculation was evaluated.

## 3. Results and Discussion

### 3.1. Influence of Raw Material Concentration and Solvents Used on the Quality of Propolis Extracts

According to the data of the literature, different concentrations (2.5%, 5%, and 10% propolis) of water and water-free extracts of propolis were made [13, 14, 17]. Evaluation of the effect of solvent and concentration of propolis on amount of phenolic compounds was performed.

The data presented in Table 1 shows that when propolis concentration increases, the total amount of phenolic compounds in extracts increases. Statistically significant difference of means according to Student's *t*-test between 2.5% and 5% water extracts of propolis was determined, *P* = 0.032 (*P* < 0.05) as well as between 5% and 10% extracts, *P* = 0.03. Statistically significant difference between studied ethanol extracts of propolis was determined (*P* < 0.05). When a 70% ethanol as solvent was used, the highest amount of phenolic compounds was released from 5% propolis extract, and the lowest amount from 10% propolis extract. More phenolic compounds were released from ethanolic extracts compared with water extracts of propolis. Statistical significant differences between studied propolis solutions in propylene glycol were determined. The highest amount of phenolic compounds was found in 10% propolis extract. When compared with water extracts, the amount of phenolic compounds was significantly higher but lower if compared with ethanolic extracts. According to the data [18, 19], to manufacture propolis extract, three-solvent system was used. The data shows that the lower extract concentrations, the higher amount of phenolic compounds is. The highest content of phenolic compounds was released from 2.5% propolis extract, and the lowest from 10%. The difference between 2.5% and 5% propolis extracts, *P* = 0.06 and between 5% and 10%, *P* = 0.05 was not statistically significant, and between 2.5% and 10%—*P* = 0.006 (*P* < 0.05)—difference was statistically significant. The results of the studies showed that the amount of phenolic compounds in propolis extracts depended not only on raw material concentration but also on solvent used in extraction process. Moreover, the data of the studies illustrates that during extraction process a big marginal layer develops and diffusion does not occur, suggesting that technological factors improving diffusion should be introduced [17].

### 3.2. Effect of Stirring on the Quality of Propolis Extracts

In order to improve the extraction of active substances, extracts with three-solvent system and propylene glycol propolis extracts were stirred with magnetic stirring. Stirring has been performed for 3 h; samples for the analysis of phenolic compounds were taken after 1 h, after 2 h, and after 3 h of stirring, respectively.

**Figure 1:**
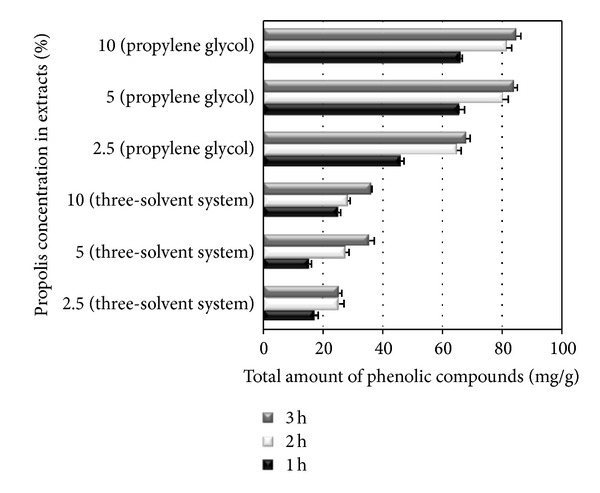
Effect of stirring ontotal amount of phenolic compounds.

The results of the study showed that stirring had an effect on the release of phenolic compounds from extracted raw material—after 1 h of stirring the highest amount of phenolic compounds was released from 2.5% propolis extract, and the lowest from 10%, and after 3 h the lowest amount of phenolic compounds was released from 2.5% propolis extract, but the highest amount from 10% propolis extract. However, a comparison of the data after two hours of stirring did not determine significant higher amounts of phenolic compounds. Differences between the total amount of phenolic compounds in the all studied propolis propylene glycol extracts and 2.5% propolis in three-solvent system stirred for 2 and 3 h were not statistically significant (*P* > 0.05).  Figure 1 shows an increase of phenolic compounds during stirring. It suggests that the optimal time to stir is 2 h. Also was established that the most suitable extraction method for propolis solution in solvent mixture is maceration. The higher amount of phenolic compounds was in propolis solution in three-solvent system produced using the method of maceration than in propolis solution prepared using stirring.

### 3.3. Influence of Temperature and Stirring on Propolis Extracts

To evaluate the influence of temperature, 5% propolis solutions in propylene glycol were used. Stirring was performed at different temperatures (40, 50, and 60°C), time of stirring—2 h. For the analysis, 5% propolis solutions in propylene glycol were used.

**Figure 2:**
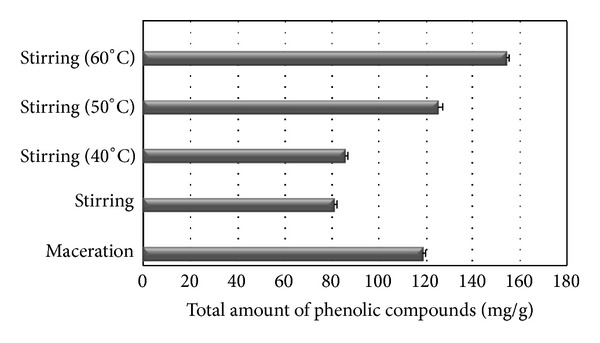
Influence of temperature on total amount of phenolic compounds in 5% propolis propylene glycol extracts.

The results showed that the lowest amounts of phenolic compounds were released when solutions were stirred for 2 h at 40°C temperature—85.6 ± 0.56, at 50°C temperature—125.2 ± 0.71 (*P* < 0.05, Student's *t*-test), and the highest content was observed when stirred for 2 h at 60°C temperature—153.8 ± 0.94 (*P* < 0.05, Student's *t*-test). It suggests that an increase of temperature has an influence on the amount of phenolic compounds. When comparing extracts obtained at different temperature, significant differences between the amounts of active substances were determined. The most effective release was occurring when 60°C was maintained. Investigation showed that maceration method is appropriate for preparing the propolis propylene glycol extracts. However, stirring at 50°C and higher temperature were more effective than maceration (*P* < 0.05, Student's *t*-test) (Figure 2).

### 3.4. Determination of Microbiological Activity of Propolis Solutions

**Figure 3:**
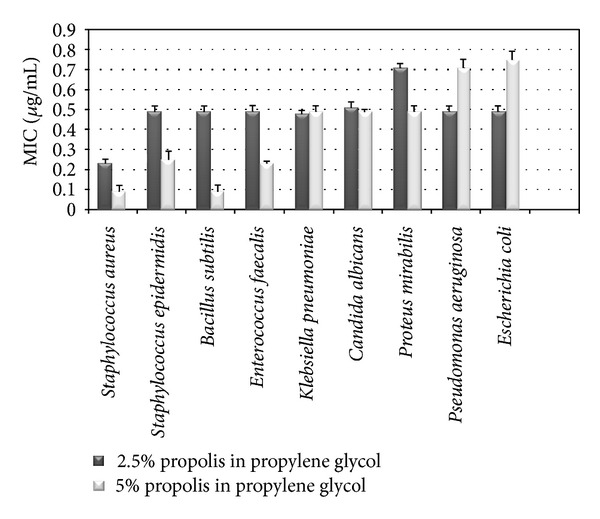
Microbiological activity of propolis phenolic compounds in propylene glycol solutions.

The results of the study showed that water extracted propolis and propolis solution in three-solvent system (water-ethanol-propylene glycol) were not effective against the studied strains of microorganisms. Ethanol 2.5% propolis extracts were more effective against the studied microorganisms compared with other investigated extracts. The result showed that the growth of *Klebsiella pneumoniae* was most resistant to the effect of propolis ethanol extract and the MIC of phenolic compounds was 0.5 *μ*g/mL. Gram-negative bacteria *Escherichia coli, Pseudomonas aeruginosa,* and *Proteus mirabilis* were more sensitive, the determined MIC 0.28 *μ*g/mL, compared to gram-positive bacteria. The MIC for the growth of *Staphylococcus aureus* was 0.17 *μ*g/mL, and for *Enterococcus faecalis*—0.2 *μ*g/mL. The most sensitive microorganisms to be studied ethanol extracts were *Candida albicans* and *Bacillus cereus, Bacillus subtilis*—the MIC was 0.06 *μ*g/mL. Propolis solutions in propylene glycol showed the greatest effect against *Staphylococcus aureus, *and* Bacillus subtilis* (Figure 3).

These solutions were also active against the other studied microorganisms (Figure 3). The studies of antimicrobial activity of propolis extracts showed that solvents had the influence on microbiological activity of propolis extracts—water extracted propolis solutions did not inhibit the growth of the studied microorganisms. The technology of this solution and evaluation of composition require additional researches.

## 4. Conclusions

Crude propolis concentration has an influence on the amounts of phenolic compounds in propolis extracts when water, ethanol, or propylene glycol are used in the process of extraction. The highest content of phenolic compounds was determined in solutions containing 10% propolis extracts, and the lowest amounts—2.5% propolis extracts. The results revealed that water used in the process of extraction extracts the lowest amount of phenolic compounds from crude propolis, ethanol—the highest amount, and propylene glycol ranks the middle position.

It is determined that technological parameters (stirring, temperature) contribute to the content of phenolic compounds when propylene glycol as solvent was used. The highest amounts of phenolic compounds was determined when temperature was 60°C, and stirring time—2 h.

The studies showed that higher content of active substances is obtained when ethanol was used. However, the data also demonstrated that both solvents used were suitable for crude propolis extraction because propylene glycol extracted propolis solutions had antimicrobial activity. Since propylene glycol is nonvolatile and contains no water, it may be widely used as solvent in manufacturing of propolis solutions.

## Figures and Tables

**Table 1:** Total amount of phenolic compounds expressed as FAE mg/g in propolis extracts. Data presented as mean ± SD, *n* = 3.

	Solvents	Total amount of phenolic compounds (mg/g)		
		Propolis concentration		
		2.5%	5%	10%
Propolis extracts	Water	14.4 ± 0.22	17.0 ± 1.12	19.6 ± 0.93
	Ethanol	167.5 ± 2.78	175.6 ± 1.89	115.4 ± 2.20
	Propylene glycol	97.9 ± 1.23	118.6 ± 1.78	171.4 ± 2.54
	Three-solvent system	85.4 ± 1.65	82.5 ± 1.36	73.7 ± 1.32
